# Performance evaluation methods for improvements at post-market of artificial intelligence/machine learning-based computer-aided detection/diagnosis/triage in the United States

**DOI:** 10.1371/journal.pdig.0000209

**Published:** 2023-03-08

**Authors:** Mitsuru Yuba, Kiyotaka Iwasaki

**Affiliations:** 1 Cooperative Major in Advanced Biomedical Sciences, Joint Graduate School of Tokyo Women’s Medical University and Waseda University, Waseda University, Tokyo, Japan; 2 Department of Modern Mechanical Engineering, School of Creative Science and Engineering, Waseda University, Tokyo, Japan; 3 Department of Integrative Bioscience and Biomedical Engineering, Graduate School of Advanced Science and Engineering, Waseda University, Tokyo, Japan; 4 Institute for Medical Regulatory Science, Waseda University, Tokyo, Japan; University of Cagliari: Universita degli Studi Di Cagliari, ITALY

## Abstract

Computer-aided detection (CADe), computer-aided diagnosis (CADx), and computer-aided simple triage (CAST), which incorporate artificial intelligence (AI) and machine learning (ML), are continually undergoing post-market improvement. Therefore, understanding the evaluation and approval process of improved products is important. This study intended to conduct a comprehensive survey of AI/ML-based CAD products approved by the U.S. Food and Drug Administration (FDA) that had been improved post-market to gain insights into the efficacy and safety required for market approval. A survey of the product code database published by the FDA identified eight products that were improved post-market. The methods used to evaluate the performance of improvements were analysed, and post-market improvements were approved with retrospective data. Reader study testing (RT) or software standalone testing (SA) procedures were conducted retrospectively. Six RT procedures were conducted because of modifications to the intended use. An average of 17.3 readers (minimum 14, maximum 24) participated, and the area under the curve (AUC) was considered the primary endpoint. The addition of study learning data that did not change the intended use and changes in the analysis algorithm were evaluated by SA. The average sensitivity, specificity, and AUC were 93% (minimum 91.1, maximum 97), 89.6% (minimum 85.9, maximum 96), and 0.96 (minimum 0.96, maximum 0.97), respectively. The average interval between applications was 348 days (minimum –18, maximum 975), which showed that the improvements were implemented within approximately one year. This is the first comprehensive study on AI/ML-based CAD products that have been improved post-market to elucidate evaluation points for post-market improvements. The findings will be informative for the industry and academia in developing and improving AI/ML-based CAD.

## Author summary

Computer-aided detection (CADe), computer-aided diagnosis (CADx), and computer-aided simple triage (CAST) utilising artificial intelligence (AI) and machine learning (ML) are under development around the world. AI/ML-based CAD is continually undergoing post-market improvement using real-world data collected from clinical sites. Therefore, regulatory authorities such as the U.S. Food and Drug Administration (FDA), the Ministry of Health, Labour, and Welfare (MHLW) and the Pharmaceuticals and Medical Device Agency (PMDA) in Japan, and the European Medicines Agency (EMA) in Europe are investigating appropriate regulation systems for AI features. Here, we investigated AI/ML-based CAD products that have been improved post-market by surveying a product code database published by the FDA. Eight products that have been modified after marketing were identified. We found that post-market improvements that did not change the intended use were approved after evaluation by a standalone software test. The findings will be informative for the industry and academia in developing and improving AI/ML-based CAD.

## Introduction

Computer-aided detection (CADe), computer-aided diagnosis (CADx), and computer-aided simple triage (CAST) incorporating artificial intelligence (AI) and machine learning (ML) have attracted considerable attention for increasing diagnostic accuracy and efficient clinical practice [1,2]. However, because AI/ML-based CAD is a novel medical technology, regulatory authorities such as the U.S. Food and Drug Administration (FDA), the Ministry of Health, Labour, and Welfare and the Pharmaceuticals and Medical Device Agency (PMDA) in Japan, and the European Medicines Agency (EMA) in Europe are investigating appropriate regulation systems for AI features.

The challenges in developing AI/ML-based CAD regulations are (1) the complexity in determining medical device applicability and (2) inadequate consensus on the appropriate regulatory framework for frequent post-market improvements. To address these challenges, the FDA has been developing regulatory policies for Software as a Medical Device (SaMD), including AI/ML-based CAD, since the 21^st^ century Cures Act was passed in December 2016 [3,4]. Furthermore, the FDA announced its ‘Digital Innovation Action Plan [5]’ to proceed with the 21^st^ Century Cures Act in 2017. The Act includes (1) developing guidelines for SaMD regulations, (2) piloting the pre-certification programme [6], and (3) strengthening the digital health unit at the Center for Devices and Radiological Health [7]. Several action plans have already been implemented. The FDA issued six guidelines on applicability for (1) general wellness products [8], (2) medical device data systems [9], (3) off-the-shelf software [10], (4) clinical decision support software [11], (5) mobile medical application [12], and (6) medical device accessories [13] in 2019.

Although the FDA is the leading regulatory authority for AI/ML-based CAD, considerable uncertainty remains regarding the evaluation methods for post-market improvement. The evaluation methods for AI/ML-based CAD devices can be classified into standalone software testing and reader study testing. Standalone software testing (SA) is defined as a performance test of the AI-only using test data that are collected retrospectively. Reader study testing (RT) is defined as a performance test that evaluates the interaction of AI with physicians on diagnostic or detection accuracy. The advantage of SA is the cost and time saving compared to RT because there is no need to recruit readers for performance evaluation. However, it cannot be used to evaluate performance in clinical practice, usability, and the affection of AI assistance. RT can be performed not only prospectively but also retrospectively using previously collected images.

A recent study on the systematic analysis to clarify the test design of AI/ML-based CAD at pre-market demonstrated that CAST was approved based on SA, whereas CADe and CADx were approved based on RT in the U.S.[14] However, no studies have been conducted to determine how post-market improvement should be evaluated. Therefore, the lack of predictability of whether post-market improvements should be evaluated using SA or RT is an obstacle for start-ups and other companies developing medical devices based on AI technology.

This study aimed to (1) investigate the guidelines for evaluating the performance of CAD in the U.S., thereby clarifying the regulation system, and (2) clarify whether post-market improvement can be evaluated using SA or RT to improve transparency of requirement.

## Methods

### Guidelines for performance evaluation

The guidelines on ‘Digital Health’ published by the FDA were obtained from the FDA website (accessed June 20, 2022). In total, 23 guidance documents issued since 2005 [4,8–13,15–30] were identified including the draft versions.

### Data sources of AI/ML-based medical devices

**Fig 1:**
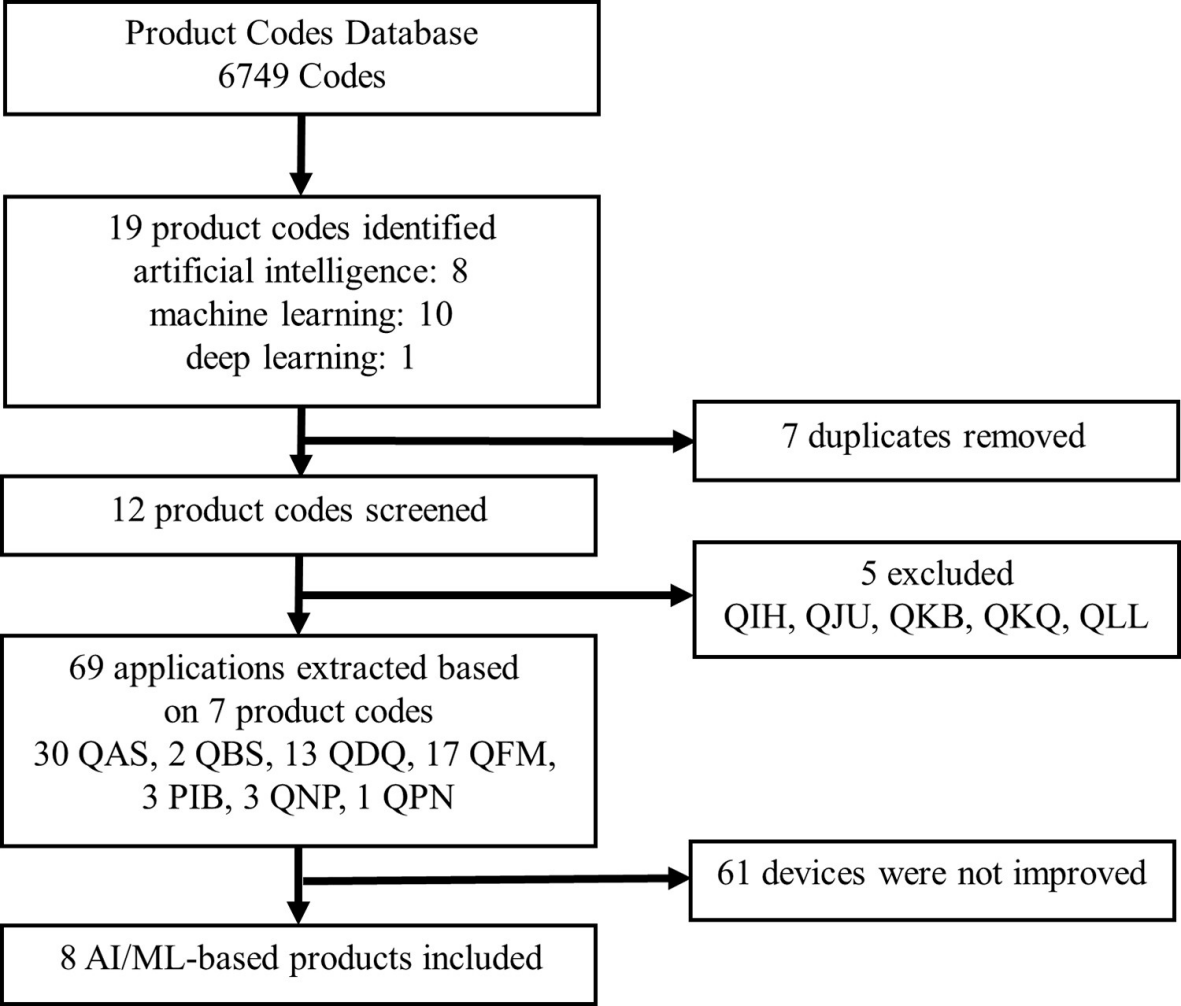
Flowchart for the extraction of AI/ML-based CAD devices with post-market improvements in the United States.

AI/ML-based CAD data were obtained from the FDA product code database (Fig 1). As of June 1, 2022 (the date from which devices were selected), 6749 product codes have been listed. Using search keywords, such as AI, ML, and deep learning, 19 product codes were identified (eight for AI, ten for ML, and one for deep learning). Among the 19 product codes, seven duplicates were removed, and five others were excluded after screening (excluding codes that did not correspond to triage, notification, detection, or diagnosis). The final seven product codes encompassed 69 devices in total. Of these, four were granted De Novo clearance and 65 were granted 510(k) clearance (no pre-market approval). Finally, eight products with the same product name, but resubmitted with post-market improvements, were included. The details of the screening and selection processes are shown in Fig 1. Information on De Novo classification requests, decision summaries, and a 510(k) summary of AI/ML-based CAD approved in the U.S. was collected. The retrieved information included: (1) the device name, (2) intended use, (3) description of change, (4) application category, (5) submission date, (6) approval date, (7) study design, (8) test case, and (9) performance (sensitivity, specificity, and area under the curve (AUC)). For each product, we also searched for additional studies via PubMed and Google scholar on how efficacy and safety have been evaluated.

### Statistics

The mean values of the test cases’ performance (sensitivity, specificity, and AUC) extracted from the 510(k) summary and De Novo classification requests and decision summaries were calculated as average (minimum–maximum) using Excel (Microsoft Corporation). The submission interval was calculated as the number of days from the date of approval to the date of reapplication, i.e., submission date–approval date.

## Results

### Policy for performance evaluation

**Fig 2:**
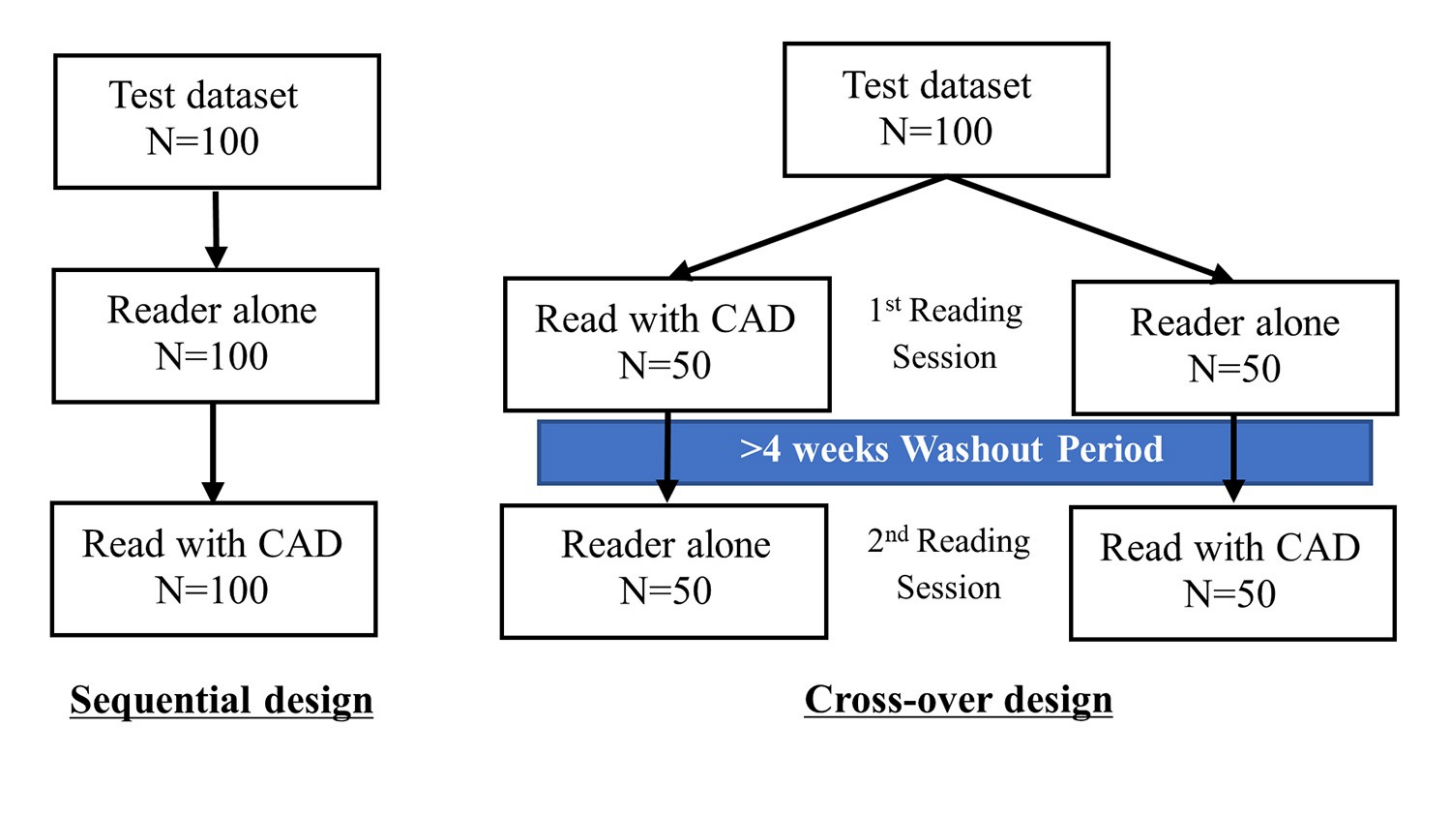
Overview of sequential design (left) and cross-over design (right).

The FDA published ‘Clinical Performance Assessment: Considerations for Computer-Assisted Detection Devices Applied to Radiology Images and Radiology Device Data in Premarket Notification 510(k) Submission [18]’ in 2012. This guideline states that CAD products used as second readers can be evaluated using sequential design (Fig 2) in which physicians first read all the test images without CAD assistance. The CAD analysis results are then referenced. CAD products used as concurrent readers, according to which, from the beginning, the doctor refers to the CAD analysis results for reading, recommend a cross-over design. In the cross-over design, the test dataset is randomly categorised into two groups, one group to be read without CAD and the other group to be read with CAD assistance for the first time. To evaluate the effectiveness of CAD assistance, after a four-week washout period, participants assigned to the group without CAD at the first reading read with CAD, and those assigned to the group with CAD at the first reading read without CAD.

Furthermore, the FDA recommends the multiple-reader-multiple-case (MRMC) protocol in which data obtained from multiple patients are read by multiple readers. However, although conducting the study under the MRMC protocol is statistically credible, the FDA does not consider this to be the case in situations where it is difficult to have multiple readers for a single patient’s data; for example, when conducting a prospective study.

In the same year, the FDA published ‘Computer-Assisted Detection Devices Applied to Radiology Images and Radiology Device Data-Premarket Notification 510(k) Submissions [19]’, which details the guidelines for evaluating software. This guideline recommends that changes or modifications to existing CAD products should be evaluated on a software standalone basis to demonstrate that the changes are substantially equivalent. Furthermore, in a software standalone evaluation, if a generic product is manufactured, it should be compared with the predicate device using the same dataset. The details of the dataset (number of facilities, radiological examination conditions, and modalities used) and its composition (number of cases and pathological conditions) should be explained when conducting a reader study testing (RT). This is the only guideline that mentions the definition of computer-aided simple triage (CAST).

The FDA proposed the Good Machine Learning Practice (GMLP) framework in April 2019 [31]. According to the GMLP, the SaMD updates can be classified into three categories: (1) performance, (2) data used, and (3) intended use. To accommodate such frequent updates, the FDA has defined SaMD pre-specifications (SPS) and the algorithm change protocols (ACP). SPS indicates the range of possible changes to AI/ML medical devices, whereas ACP indicates machine learning models, data collection, and management methods. If it is a post-marketing update, the FDA determines the application category by reviewing its effect on SPS and ACP.

### Approved or cleared AI/ML-based CAD improved at post-market

AI/ML-based CAD products marketed in the U.S. were drawn from the product codes published by the FDA, and reapplications were submitted for eight products that were modified after marketing (Table 1). Details of the evaluation method are shown in Table 2.

**Table 1:** Characteristics of the 8 devices improved at post-market in the USA. RT, Reader study testing; SA, Software standalone testing; DL, Deep learning. ※Submission interval = Submission Date -Approval Date.

#	Device name	Intended use	Aim of change	510(k) /De Novo	Submission date	Approval date	Submission interval※	Evaluation method	AI Algorithm
**1**	Transpara 1.3	To detect breast cancer for 2D DM	N/A	510(k)	6.27.2018	11.21.2018	N/A	RT	DL
	Transpara 1.5	To detect breast cancer for 2D DM	Addition of Fujifilm’s DM as supported system	510(k)	8.22.2019	12.10.2019	274	SA	DL
	Transpara 1.6	To detect breast cancer for 2D DM and DBT	Addition of DBT as supported system	510(k)	11.22.2019	3.5.2020	–18	RT	DL
	Transpara 1.7	To detect breast cancer for 2D DM and DBT	Addition of Fujifilm’s DBT as supported system	510(k)	2.10.2021	6.2.2021	342	SA	DL
**2**	ProFound V2.0	To detect and diagnose breast cancer for DBT	N/A	510(k)	8.31.2018	12.6.2018	N/A	RT	DL
	ProFound V2.1	To detect and diagnose breast cancer	Addition of Siemens’s DBT as supported system	510(k)	7.26.2019	10.4.2019	232	SA	DL
	ProFound V3.0	To detect and diagnose breast cancer	To improve specificity	510(k)	12.29.2020	3.12.2021	452	SA	DL
**3**	MammoScreen	To detect breast cancer	N/A	510(k)	10.4.2019	3.25.2020	N/A	RT	DL
	MammoScreen 2.0	To detect breast cancer	Addition of DBT as supported system	510(k)	5.19.2021	11.26.2021	420	RT	DL
**4**	Accipiolx	To triage and notify of intracranial haemorrhage	N/A	510(k)	8.10.2018	10.26.2018	N/A	SA	DL
	Accipiolx	To triage and notify of intracranial haemorrhage	To improve performance by changing algorithm from ML to CNN	510(k)	5.15.2020	8.7.2020	567	SA	DL
**5**	Viz ICH	To triage and notify of intracranial haemorrhage	N/A	510(k)	12.30.2019	3.18.2020	N/A	SA	AI
	Viz ICH	To triage and notify of intracranial haemorrhage	Addition of GE Healthcare’s non-contrast CT as supported system	510(k)	1.26.2021	3.23.2021	314	SA	AI
**6**	HALO	To triage and notify of large vessel occlusion	N/A	510(k)	4.1.2020	11.20.2020	N/A	SA	AI
	HALO	To triage and notify of large vessel occlusion	Addition of Philips’s contrast CT angiogram as supported system	510(k)	6.9.2021	7.8.2021	201	SA	AI
**7**	GI Genius	To detect colonic mucosal lesions	N/A	De Novo	9.8.2020	4.9.2021	N/A	RT	DL
	GI Genius 2.0	To detect colonic mucosal lesions	Addition of Fujifilm’s endoscopy as supported device	510(k)	6.23.2021	7.23.2021	75	SA	DL
**8**	IDx-DR	To detect and diagnose more than mild diabetic retinopathy	N/A	De Novo	1.12.2018	4.11.2018	N/A	RT	AI
	IDx-DR	To detect and diagnose more than mild diabetic retinopathy	Addition of training mode and incorporation of DICOM images etc	510(k)	12.11.2020	6.10.2021	975	SA	AI

**Table 2:** Evaluation method of the 8 devices improved at post-market in the USA. MRMC, Multi-reader multi-case; MQSA, Mammography Quality Standards Act.

#	Device name	Test design	Test case	Reader	Clinical site	Sensitivity	Specificity	AUC	CAD type
**1**	Transpara 1.3	Retrospective, MRMC, RT, fully-crossed	240 images	14 radiologists	2	N/A	N/A	0.88 (0.86)	CADe/CADx
	Transpara 1.5	Retrospective, SA	N/A	N/A	N/A	N/A	N/A	N/A	CADe/CADx
	Transpara 1.6	Retrospective, MRMC, RT, fully-crossed	240 images	18 MQSA qualified radiologists	N/A	N/A	N/A	0.863 (0.833)	CADe/CADx
	Transpara 1.7	Retrospective, SA	N/A	N/A	N/A	N/A	N/A	N/A	CADe/CADx
**2**	ProFound V2.0	Retrospective, MRMC, RT, fully-crossed	260 images	24 tomosynthesis radiologists	N/A	85 (77)	69.6 (62.7)	0.852 (0.795)	CADe/CADx
	ProFound V2.1	Retrospective, SA	694 images	N/A	N/A	N/A	N/A	N/A	CADe/CADx
	ProFound V3.0	N/A	N/A	N/A	N/A	N/A	N/A	N/A	CADe/CADx
**3**	MammoScreen	Retrospective, MRMC, RT, fully-crossed	240 images	14 radiologists	N/A	N/A	N/A	0.8 (0.77)	CADe/CADx
	MammoScreen 2.0	Retrospective, MRMC, RT, fully-crossed	240 images for each modality	34 radiologists (14 for the 2D study and 20 for the 3D study)	N/A	N/A	N/A	2D: 0.79 (0.77) 3D: 0.83 (0.79)	CADe/CADx
**4**	Accipiolx	Retrospective, SA	360 images	N/A	30	92	86	N/A	CAST
	Accipiolx	Retrospective, SA	360 images	N/A	17	97	93	N/A	CAST
**5**	Viz ICH	Retrospective, SA	261 images	N/A	2	93	90	0.96	CAST
	Viz ICH	Retrospective, SA	387 images	N/A	2	95	96	0.97	CAST
**6**	HALO	Retrospective, SA	364 images	N/A	Multi	91.1	87	0.97	CAST
	HALO	Retrospective, SA	427 images	N/A	Multi	91.3	85.9	0.97	CAST
**7**	GI Genius	Prospective, Randomised, the primary outcome was adenoma detection rate (ADR)	700 patients	6	3	54.8 (40.4)	N/A	N/A	CADe
	GI Genius 2.0	Retrospective, software standalone testing, comparison between performance of predicate device and improved device based on same test dataset	Per-frame assessment on 40 videos	N/A	N/A	86.5 (82.0)	N/A	0.796 (0.723)	CADe
**8**	IDx-DR	Prospective, MRMC, Comparison between output of IDx-DR and Expert who had been certified by the Fundus Photography Reading Center (FPRC)	819 patients	N/A	10	87	90	N/A	CADe
	IDx-DR	N/A	N/A	N/A	N/A	N/A	N/A	N/A	CADe

The initial approval for Transpara V1.3 (ScreenPoint Medical B.V.) was reviewed in November 2018 [32], and a second application was submitted in August 2019 to add FUJIFILM’s digital mammogram (DM) as a supported device [33]. Furthermore, in November of the same year, a third submission was filed to add digital breast tomosynthesis (DBT) as a supported device [34]. Subsequently, its performance was evaluated by RT. In March 2020, a fourth application was filed to add DBT manufactured by FUJIFILM [35]. All applications were submitted within a year of obtaining the 510(k). In particular, the third application for Transpara V1.6, which added DBT as a co-usable imaging device, was submitted during the V1.5 review process [33,34]. This result indicates that improvement can be introduced even during the review process (Table 1). RT was conducted at the time of the Transpara V1.3 [36] and Transpara V1.6 applications [37], and the number of images used for evaluation was standardised to 240. The numbers of readers that participated in the studies were 14 and 18, and both applications were approved after confirming that the improvement in the AUC was assisted by CAD.

No information on the test methods of Transpara V1.5 and V1.7 evaluated during software standalone testing (SA) was disclosed in the 510(k) summary (Table 2). However, V1.7 was evaluated in SA and the results were published in 2022 [38]. The performance on 15000 DM or DBT was evaluated, achieving AUC of 0.93 for DM images and 0.94 for DBT images. In addition, several studies using V1.4 were identified, although it has not been filed as product [39–41]. In a study comparing the diagnostic performance of V1.4 with 101 radiologists, the AUC of V1.4 was 0.84, while that of the radiologist was 0.814, indicating that V1.4 performed better [39].

Of the products for detecting/diagnosing breast cancer, ProFound AI Software V2.0 (iCAD Inc.) was initially approved in December 2018 [42] and then submitted again in July 2019 (V2.0 to V2.1) to add Siemens’s DBT as a co-usable device [43]. Furthermore, an application was filed in December 2020 to improve the specificity (V2.1 to V3.0) (Table 1) [44]. Performance was only evaluated by RT for the initial application, with 24 readers participating and using 260 images [42,45]. The 510(k) summary of ProFound V3.0 did not provide any detailed information on the test method, only stating that it improved specificity [44].

MammoScreen (Therapixel), which is used to detect breast cancer, was initially approved in March 2020 [46] and subsequently resubmitted in May 2021 to add DBT as a supported device [47]. With the initial approval, RT was performed, and a 510(k) was obtained (Table 1). MammoScreen 2.0 had two RTs (evaluation for DM and evaluation for DBT), each with 240 images, with 14 and 20 readers participating, respectively (Table 2).

Accipiolx (MaxQ AI Ltd.) for detecting intracranial haemorrhaging had received initial approval in 2018 [48]. The application was resubmitted again in 2020 [49] because of the subsequent change from an ML-based algorithm to a convolutional neural network-based algorithm (Table 1). The change improved Accipiolx’s sensitivity from 92% to 97% and its specificity from 86% to 93%. Three hundred and sixty cases were used to evaluate its performance. The number of clinical sites from which images were collected decreased from 30 to 17, while the number of images used to evaluate performance remained unchanged at 360 (Table 2).

Viz ICH (Viz ai Inc.), developed for triaging intracranial haemorrhage, received a 510(k) in March 2020 [50] and an application was submitted in October 2021 [51] to add GE Healthcare’s CT as a supported device (Table 1). The number of images used for evaluation was increased from 261 to 387, the sensitivity from 93% to 95%, the specificity from 90% to 96%, and the AUC from 0.96 to 0.97 (Table 2).

HALO (NICO-Lab B.V.), developed for triaging large vessel occlusion, received a 510(k) in November 2020 [52], and in June 2021, an application was submitted to add the Philips CT angiogram (Table 1) [53]. The number of images used for evaluation was increased from 364 to 427, and the sensitivity, specificity, and AUC were comparable to predicate devices (Table 2).

GI Genius (Cosmo AI), for the detection of colon mucosal lesions, received approval in a De Novo application [54], and 102 days later, an application was filed to add FUJIFILM’s endoscope as a possible concomitant device [55]. Unlike the initial application, the performance of GI Genius was evaluated by SA (Table 1). A prospective randomised controlled trial was conducted for the initial application, involving six experienced endoscopists (two for each centre, >2000 screening colonoscopies) in three centres. The primary endpoint was the adenoma detection rate (ADR), defined as the percentage of patients with at least one histologically proven adenoma or cancer. The results showed that ADR was 40.4% without CAD assistance, whereas ADR with CAD assistance improved to 54.8%, and the application was approved. At the time of improvement, comparisons were made by a per-frame evaluation of 40 videos, and the sensitivity was improved from 82% to 86.5% and AUC from 0.723 to 0.796 (Table 2).

IDx-DR (Digital Diagnostics Inc.), which identifies diabetic retinopathy, underwent minor changes including the addition of a training mode 975 days after it was approved in the De Novo application [56,57]. The evaluation performed at resubmission was SA (Table 1). At the time of the initial submission, IDx-DR analysed images taken by non-specialists at 10 primary-care sites, and the results were evaluated by comparing them with the results of specialist diagnoses. At resubmission, the evaluation only confirmed that the training mode was functioning, and no tests were conducted to evaluate performance (Table 2).

The mean interval between post-approval and reapplication for the eight products that underwent post-market improvement was 348 days (minimum –18, maximum 975). Including the initial application, six retrospective RTs (MammoScreen 2.0’s 2D and 3D study were counted as one trial each) were conducted, and the mean number of participating readers was 17.3 (minimum 14, maximum 24). The mean number of images used for evaluation was 243 (minimum 240, maximum 260), and the mean AUC of was 0.83 (minimum 0.79, maximum 0.88).

There were six SAs for which details were available. The mean number of images used for evaluation was 359.8 (minimum 261, maximum 427). The mean values for sensitivity and specificity were 93% (minimum 91.1, maximum 97) and 89.6% (minimum 85.9, maximum 96), respectively, and AUC was evaluated in four studies with a mean value of 0.96 (minimum 0.96, maximum 0.97).

## Discussion

This study elucidated that (1) post-market improvements are approved with retrospective data, (2) post-market improvements that do not change the intended use are approved by evaluating through SA, and (3) products are being developed for triage.

### Evaluation for improvement at post-market

Three types of post-market improvements of medical devices using AI have been defined in the order of risk: (1) performance: clinical and analytical performance, (2) input data: they are used by the algorithm and their clinical association with the SaMD output, and (3) intended use: the intended use of the SaMD and the significance of information provided by the SaMD on the state of the healthcare situation or condition [31]. Considering the cases of change obtained in this study based on this classification, the change from Transpara V1.5 to V1.6, which added DBT and a mammography unit as supported devices that can be analysed, and the change to MammoScreen 2.0 correspond to (3) ‘intended use’. Therefore, RT was used as the evaluation method for reapplication because it has the highest risk change. The average number of readers required to conduct RT was 17, and therefore a sufficient adjuvant effect independent of the operator’s experience must be demonstrated. AUC is the first choice as the primary endpoint.

The changes from Transpara V1.3 to V1.5, V1.6 to V1.7, ProFound V2.0 to V2.1, the improvement of GI Genius, improvement of Viz ICH, and the improvement of HALO with additional manufacturers are less risky than changes in the intended use. Because these changes are related to (2) input data, we believe that SA was used to evaluate and approved by confirming substantial equivalence. The GI Genius case was based on 40 videos; however, it was a frame assessment. Thus, we believe it was considered that endoscopy is a video-based examination, unlike CT.

The improvement of Accipiolx from ML-based algorithms to algorithms that use deep learning, the improvement of ProFound V2.1 to V3.0 with improved specificity, and the improvement of IDx-DR with added training mode functions fall under (1) performance. Improvements that fall under the (1) and (2) categories are considered to be approved if substantial equivalence can be detailed by conducting a performance comparison with the predicate device in SA.

AI/ML-based CAD, which is trained based on limited training data, can be used to collect various types of data continuously after approval. Therefore, additional modalities and devices from various development manufacturers will be added within approximately a year. In this study, the developmental life cycle of AI/ML-based medical devices is considerably shorter than that of conventional medical devices, especially because of the cases in which applications were filed again even while under review. In particular, the Transpara series incorporated in this study had undergone a performance evaluation with a larger amount of data at post-market [38,39,58]. Performance evaluation of V1.3 on 310 Japanese women showed that the AUC for readers without AI assistance was 0.816, whereas V1.3 alone showed a lower value of 0.706, demonstrating the necessity of improvement [58]. Therefore, a crucial feature of AI/ML-based CAD is that it can be quickly upgraded to resolve issues that have been identified in real-world clinical practice.

In this study, we performed a keyword search based on product codes and identified that, of the 12 product codes, six codes contain AI and ML, and one code contains both AI and DL. This implies that the FDA does not make a distinction based on AI/ML/DL. The notation AI/ML is present in the guidance issued by the FDA [31,59]. Furthermore, the FDA guidance indicates that ML and DL are one of the AI technologies. Therefore, the FDA is supposed to categorize and regulate based on the intended use, such as target disease and type of CAD.

### Applicability of computer-aided simple triage

**Fig 3:**
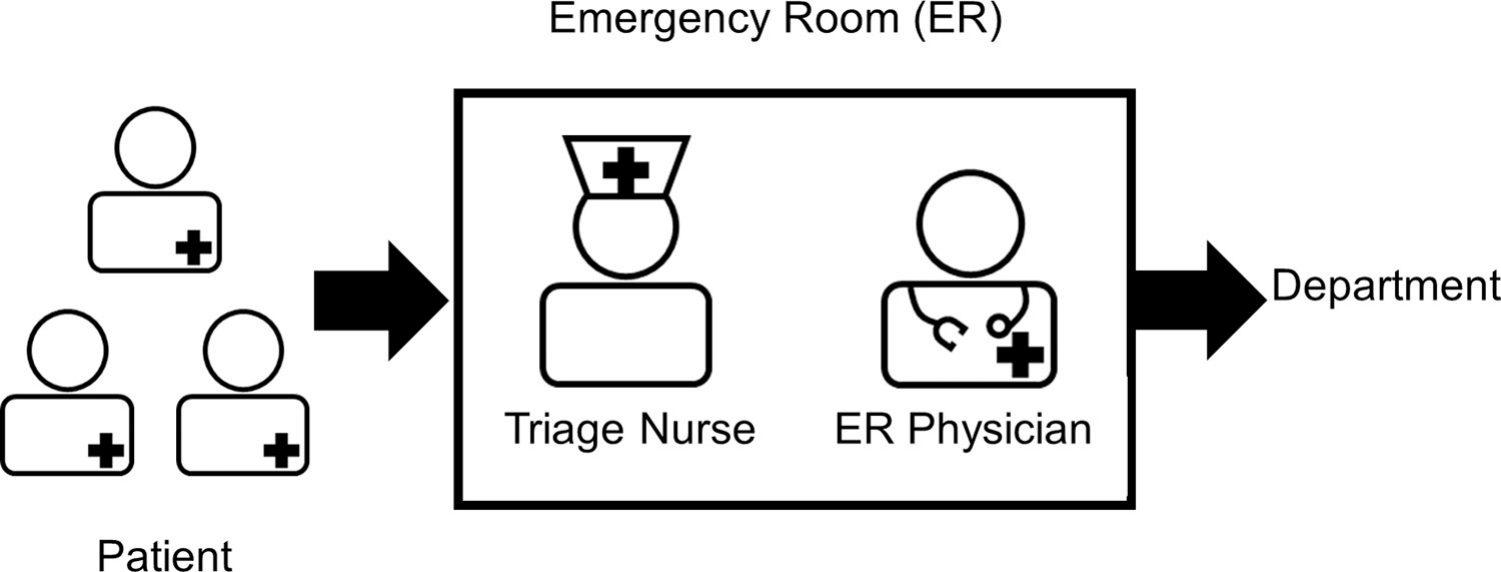
Overview of the emergency room system in the United States.

The emergency medical system has an important role in accelerating development and application of CAST as a medical device. In the U.S., the emergency room (ER) system is mainly used (Fig 3). In this system, all emergency patients are accepted at the ER where they are examined by ER specialists or triage nurses. After initial diagnoses and treatment, patients are sent to the appropriate departments based on their medical conditions. ERs operate 24 hours per day, 365 days a year, and medical staff generally work in three shifts. A problem of this system is that ERs become crowded with large influxes of patients and accepting additional cases becomes difficult. Therefore, CAST is being studied to alleviate congestion and perform rapid diagnoses. A previous study on the implementation of AI/ML-based CAD approved by the FDA in the ER indicated that numerous products are available for immediate use in emergency medicine [60]. The main targets of AI/ML-based CAD in ERs are stroke, followed by intracranial haemorrhage, spine injury, and other circumstances in which a delay of a few minutes could worsen the prognosis. AI/ML-based CAD effectively supports this strategy. In the U.S., the reason for CAST’s introduction is the urgent need to reduce congestion. This method is a solution in ER-based emergency medical care systems, and the life-preservation rate can be improved by efficiently passing patients to the appropriate departments. Furthermore, although ER specialists may perform the initial diagnosis and treatment, they are not specialists in every department. Therefore, the introduction of CAST and its notification to specialists in the relevant departments when a patient with an extreme illness is detected enables the rapid initiation of specialist treatment.

### Limitations

This study has the following limitations: (1) We focused only on U.S. regulations, (2) we only extracted AI/ML-based CAD that could be searched using the product code. The EMA and Japan’s PMDA are regulatory authorities, as is the FDA. However, these agencies are yet to prepare a system allowing the comprehensive identification of products that have obtained the CE (European conformity) mark. The PMDA does not have a public database; therefore, this study was limited to U.S. approval cases only. Because the present systematic surveillance is qualitative, statistical analysis could not be performed. Nevertheless, the findings from the present systematic surveillance may provide valuable insights into how to improve AI/ML-based CAD products for regulatory approval in post-market.

## Conclusion

This is the first comprehensive study on AI/ML-based CAD products that have been improved post-market, to elucidate evaluation points for post-market improvements.

It was revealed that (1) post-market improvements are approved with retrospective data, (2) post-market improvements that do not change the intended use are approved by evaluation using SA, and (3) products are being developed for triage. Industry, regulatory bodies, and academia should continuously discuss the implementation of regulations that exploit the characteristics of AI/ML-based medical devices. These regulations should be developed on the premise of post-market improvement to reduce the burden on healthcare professionals and ensure patient safety.

The findings of this study will contribute to promoting post-market improvement with understanding regulations of AI/ML-based CAD that ensures the efficacy, safety, and quality of these products.
